# Self-Trained Supervised Segmentation of Subcortical Brain Structures Using Multispectral Magnetic Resonance Images

**DOI:** 10.1155/2015/764383

**Published:** 2015-10-25

**Authors:** Michele Larobina, Loredana Murino, Amedeo Cervo, Bruno Alfano

**Affiliations:** ^1^Istituto di Biostrutture e Bioimmagini, CNR, Via Tommaso De Amicis 95, 80145 Napoli, Italy; ^2^Istituto per le Applicazioni del Calcolo “Mauro Picone”, CNR, Via Pietro Castellino 111, 80131 Napoli, Italy; ^3^Dipartimento di Scienze Biomediche Avanzate, Università “Federico II”, Via Sergio Pansini 5, 80131 Napoli, Italy

## Abstract

The aim of this paper is investigate the feasibility of automatically training supervised methods, such as *k*-nearest neighbor (*k*NN) and principal component discriminant analysis (PCDA), and to segment the four subcortical brain structures: caudate, thalamus, pallidum, and putamen. The adoption of supervised classification methods so far has been limited by the need to define a representative training dataset, operation that usually requires the intervention of an operator. In this work the selection of the training data was performed on the subject to be segmented in a fully automated manner by registering probabilistic atlases. Evaluation of automatically trained kNN and PCDA classifiers that combine voxel intensities and spatial coordinates was performed on 20 real datasets selected from two publicly available sources of multispectral magnetic resonance studies. The results demonstrate that atlas-guided training is an effective way to automatically define a representative and reliable training dataset, thus giving supervised methods the chance to successfully segment magnetic resonance brain images without the need for user interaction.

## 1. Introduction

Brain tissue classification is an important topic in magnetic resonance (MR) brain image analysis. In the last years, the major tissues such as gray matter (GM), white matter (WM), and cerebrospinal fluid (CSF) have been largely studied with particular attention on volumetric variations due to the aging process or the evolution of degenerative diseases. In the recent years, a growing interest in the classification of minor brain structures has emerged [1–5]. The segmentation of the minor brain structures presents a higher degree of difficulty due to a variable and often lower contrast between these structures and adjacent tissues, which limits intensity-based classification even in the presence of multispectral data [6–8].

Supervised classification methods have shown very good results for the segmentation of MR brain images; they require the construction of a training dataset to learn how to classify new data. This represents a time consuming and expensive task, which can be achieved only by expert operators who should manually label a certain number of MR studies. Moreover, in this way, the methods tend to perform well only for studies acquired using the same acquisition protocol of the training dataset. Nowadays, the huge amount of MR brain data generated in large-scale clinical studies has prompted the development of automatic classification which should avoid or minimize the human intervention. The aim of the present work is to develop and test a completely automatic procedure to build training datasets for the classification of MR brain images using supervised methods. The method is based on the use of probabilistic atlases to guide the selection of a training dataset within the same MR study to be classified.

The idea to automatically train a supervised classification scheme using an atlas was first presented by Cocosco et al. and later by Vrooman et al. [9–11]. These authors focused primarily on segmenting brain MR images into WM, GM, CSF, and white matter lesions. They used a* k*-nearest neighbor (*k*NN) working only with MR intensity information for segmentation and lesion detection. A combination of spatial and local features using a* k*NN classifier for the segmentation of the caudate was presented by Arzhaeva et al. with a manually defined training set [12]. Various different approaches have been followed so far to segment subcortical regions but, to the best of our knowledge, the potential classification ability of* k*NN and discriminant analysis methods were not fully explored. Recently the MICCAI 2012 Gran Challenge Workshop focused on the multiatlas labeling segmentation approach, presenting the results obtained by numerous research groups using a common dataset of 35 T1 MR images from the publicly available OASIS database [13]. This dataset that contains 14 manually segmented subcortical structures to be used as reference does not include multispectral data [14]. Among the most widely used and freely available software we find two automatic methods: FreeSurfer (Martinos Center for Biomedical Imaging, Charlestown, Massachusetts, USA) [15] and FSL-First (Centre for Functional Magnetic Resonance Imaging of the Brain, Oxford, UK) [16, 17]. Both of these software packages that need only T1w images to achieve the segmentation are often used for comparison in the evaluation of new developed methods.

In this paper we present results from* k*NN and principal component discriminant analysis (PCDA) segmentation methods. The methods utilize an atlas-guided automatic training selection and work with a combination of voxel spatial locations and intensities using multispectral data. By using nonlinear spatial registration of the tissue probability atlases to the subject, the training set is tailored to the target study. The spatial a priori information of the atlas guides the choice of a representative number of voxels that serve as intensity and spatial location sample information for each of the four structures to be segmented: caudate, thalamus, pallidum, and putamen.

We present and discuss experiments conducted on 20 real studies selected from two publicly available sources of multispectral magnetic resonance studies. Fourteen studies from the IXI database of the Imperial College in London consist of T1-, T2-, and PD-weighted images. The remaining six studies (3 subjects scanned twice) were taken from the Kirby21 database of the Kirby Research Center in Baltimore, from which we selected T1- and T2-weighted and fluid attenuation inversion recovery (FLAIR) images. The scan-rescan data of three subjects served two purposes: first, to test the reproducibility of segmentation methods in providing volume estimates in subjects who were scanned twice within the same day and, second, to demonstrate that the automatically trained segmentation methods considered can work well with other acquisition sequences and can hence be thought as sequence independent. Differences in the behavior of the methods with respect to training are described. The relative performance of the autotrained* k*NN and PCDA methods is shown and discussed. The accuracy of both methods was assessed on the fourteen IXI datasets based on visual analysis of the segmentation results by an expert observer. These results are also compared with those obtained with the FSL-First software in terms of volume differences and percent of volume overlap.

## 2. Materials and Methods

Two supervised segmentation methods were considered in this work.

*(i) k-Nearest Neighbor (kNN).* The implemented* k*NN classifier was carried out using the* knnclassify* function of the Matlab package (The Mathworks, Inc.). The algorithm combines intensity and spatial features as described in the work of Anbeek et al. [18]. Briefly, for each brain voxel to be classified the three spatial coordinates and the multispectral intensity information (the number of components depends on how many different contrast-weight MR images have been considered as input) have been considered. Based on these features, each voxel was assigned to the brain tissue class that, according to a distance measure, receives the largest vote amongst the* k*-nearest neighbor belonging to the training [19]. For all the experiments we considered the Euclidean distance and a value of *k* = 40. The value of* k* has been set on the base of the observations reported by other research groups for the brain tissue segmentation task [11, 20] and our numerical experiments. 

*(ii) Principal Component Discriminant Analysis (PCDA).* The segmentation algorithm, belonging to the family of discriminant analysis methods [21], was implemented using an in-house software written in Matlab (see Appendix B for a description of the function). Starting from the training, the method performs a nonparametric estimate of tissue's probability density functions. The original components were transformed into principal components prior to estimating the probability densities. Each brain voxel was then assigned to one of the brain tissues applying the Bayes decision rule. Intensity values (the number of components depends on how many different contrast-weight MR images have been considered as input) and spatial coordinates of the voxels have been considered by the classifier as discriminant features.

For both segmentation methods, all feature values were shifted and rescaled to have zero mean and unit variance.

### 2.1. MRI Data

We used two different datasets of images for method set-up and evaluation, for a total of 20 MRI studies.

*(1) Image Dataset I.* Fourteen subjects with no evidence of pathology, age range: 25–82 (6 M, 8 F), were selected from the publicly available IXI database (see Appendix A). Seven subjects were acquired at 1.5T and seven at 3T, in two different hospitals. The data from each subject consists of T1w, T2w, and PDw images. The scanning parameters for the 1.5T studies were T1w (TR/TE = 9.8/4.6 ms, flip angle 8°, voxel size 0.94 × 0.94 × 1.20 mm) and PD-T2w (TR/TE = 8178/8.0 − 100.0 ms, voxel size 0.94 × 0.94 × 1.25 mm); the scanning parameters for the 3T studies were T1w (TR/TE = 9.6/4.6 ms, flip angle 8°, voxel size 0.94 × 0.94 × 1.25 mm) and PD-T2w (TR/TE = 5725/8.0 − 100.0 ms, voxel size 0.94 × 0.94 × 1.25 mm).

*(2) Image Dataset II.* Three subjects with no history of neurological disease, age range: 25–30 (2 M, 1 F), were selected from the publicly available Kirby21 database (see Appendix A). Each subject was scanned twice with a protocol from which we selected T1w, T2w, and FLAIR as input for the classifiers; thus, this dataset consists of six MR studies. Images were acquired on a 3T scanner and the scan parameters were T1w MPRAGE (TR/TE/TI = 6.8/3.1/842 ms, flip angle 8°, voxel size 1.0 × 1.0 × 1.2 mm, sense acceleration factor = 2), T2w 3D TSE (TR/TE = 2500/287 ms, voxel size 0.9375 × 0.9375 × 1.0 mm), and FLAIR (TR/TE/TI = 8000/331/2400 ms, voxel size 0.417 × 0.417 × 0.55 mm, sense acceleration factor = 2).

### 2.2. Preprocessing

Before starting the training and subsequent classification step, the MR images of each subject were coregistered and/or resliced when necessary. With* Image dataset I*, where the T1w images were acquired in the sagittal plane and T2w and PDw in the axial plane, T1 images were resliced to the axial orientation. With* Image dataset II*, FLAIR and T2w images were coregistered and resliced to the image space of the MPRAGE images. All datasets were corrected for MR field inhomogeneity using SPM12 (http://www.fil.ion.ucl.ac.uk/spm) bias correction function with very light regularization. Then, all the tissue probabilistic atlases were reported into the Montreal Neurological Institute (MNI) space. The matrix of the nonlinear transformation that maps the MR study onto the MNI space was estimated by means of the SPM12 segment function and used to map tissue probabilistic atlases to the subject with the SPM12 inverse deformations utility.

Training was then performed for each tissue class (and for each MR contrast) as described in Training Data. These training datasets were used by the algorithms for the segmentation of the subjects intracranial brain volume after the nonbrain structures were removed using the Brain Extraction Tool FSL-BET [22].

### 2.3. Training Data

**Figure 1:**
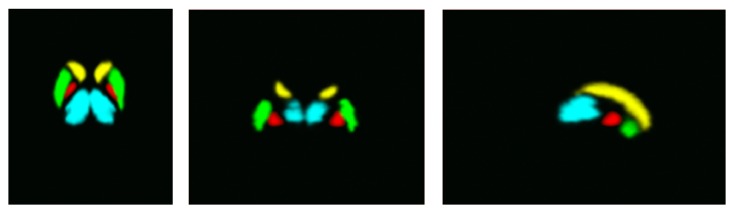
An axial, coronal, and sagittal slice of the subcortical structures probabilistic atlas: caudate (yellow), thalamus (cyan), pallidum (red), and putamen (green).

To automatically select training data from each subject, the segmentation framework presented requires a probability map for each intracranial tissue or structure to be segmented. A unique probabilistic atlas including all four subcortical tissues was constructed grouping information from the following atlases available in the literature: the International Consortium for Brain Mapping (ICBM) deep nuclei probabilistic atlas for the putamen, thalamus, and caudate [23]; and the Colin27 high-resolution single subject template to map the pallidum [24].  Figure 1 shows an axial, coronal, and sagittal slice of the obtained subcortical tissues probabilistic atlas. To discriminate subcortical structures from the underlying WM, GM, and CSF, a training is required to construct the density functions of these three major brain tissues. For this task we take into account both the ICBM452 probabilistic tissue atlas and the result of a reference segmentation method like SPM.

After the coregistration of all the probability maps to the subject (see Section 2.2), the atlas was constructed superimposing on the probability map of GM, WM, and CSF the subcortical tissue atlases in the following order: pallidum, putamen, thalamus, and caudate. Due to the heterogeneous nature of the database some overlaps of the maps can occur. In these cases a voxel is assigned to the last superimposed layer. All the atlases were initially rescaled in the range [0,1].

**Figure 2:**
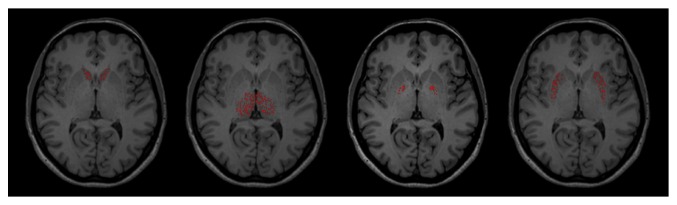
The randomly generated trainings points (in red) overlapped to a T1w axial slice for one of the studies of the* Image dataset I* (IXI002-Guys-0828). Spatial coordinates and the corresponding multispectral intensities of these points were used by the classifiers for the learning phase.

The training was then defined on the target study to be segmented in the following way. First, tissue probability maps were coregistered to the T1w of the subject and thresholded before automatically selecting the training samples for each tissue class. Four different threshold values have been evaluated in the range of 0.6 to 0.9. A threshold of 0.8 has been recognized as optimal value; lower threshold values lead to greater inaccuracy (noisy classification) while a threshold value of 0.9 may reduce too much the number of points for some of the tissues. Second, the voxels selected for training samples for each tissue class were chosen randomly.  Figure 2 shows the trainings points overlapped to an axial slice for one of the studies of the* Image dataset I*. Spatial coordinates of the training samples and the corresponding multispectral intensities were used by the* k*NN and PCDA classifiers for the learning step.

### 2.4. Processing and Analysis

Several experiments were conducted to optimize parameters and training selection and then to assess the accuracy and the robustness of the two automatically trained classifiers.

The first experiment conducted on the* Image dataset I* allowed us to (i) establish the optimal threshold for the tissue probabilistic atlases, (ii) find the optimal number of training samples to be used by the classifiers and to assess the behavior of the two methods considered with respect to the training, and (iii) realize that for the three main tissues the use of a reference segmentation as tissue probability map in place of a smoothed atlas such as the ICBM452 gives more stability and accuracy for segmentation of minor structures. After this first experiment, we focused our attention on the segmentation of subcortical structures using the segmentation of the three main brain tissues achieved with the SPM software as preprocessing. Our choice of the SPM software was because this software package was used to coregister tissue probability maps to the subject in our processing pipeline. As the SPM segmentation output is probabilistic, the same threshold value of 0.8 was applied for the training definition of GM, WM, and CSF.

The second experiment, always conducted on the* Image dataset I*, was aimed at evaluating the accuracy of the segmentation of the four subcortical structures. As a reference standard was not available for these studies, a semiquantitative evaluation of segmentation results based on visual analysis by an expert observer (Amedeo Cervo) was performed. For each MR study the visual inspection was performed slice by slice using the OsiriX software (http://www.osirix-viewer.com/), keeping the segmented structure on one series and the MR images, mainly the T1w, on another series and using the image fusion utility to view, with a user selectable fusion percentage, the segmented result overlapped to the MR signal. A score on a 5-point rating scale (very poor, poor, fair, good, and very good) was assigned for each structure and for each of the two methods,* k*NN and PCDA. From the table containing these annotations we calculated the median value and the first and third quartile for each of the four subcortical structures and for each of the two methods.

The third experiment was a reproducibility test conducted on the* Image dataset II* representing MR images from three subjects that underwent the MR scan twice in the same day. The reproducibility was evaluated by computing, for each tissue, the volume difference between the two repeated imaging sessions for both* k*NN and PCDA classifiers. The availability of FLAIR images (that condense the T2/PD information) allowed us to also investigate the response of the classifiers when only two MR contrasts instead of three (i.e., T1w and FLAIR) were considered as input.

## 3. Results

The fourteen studies of* Image dataset I* (with images weighted in T1, T2, and PD) were used for training optimization, evaluation of segmentation accuracy, and comparison with an existing method (FSL-First). The six studies of* Image dataset II* (with T1w, T2w, and FLAIR images) were used for the reproducibility test.

### 3.1. Training Optimization

The* k*NN and PCDA methods exhibit different behaviors with respect to the training. PCDA is less sensitive to the number of training samples for each class. A number of voxels corresponding to roughly the 10% of the volume of each tissue allow the PCDA to work well. No significant variations were observed by increasing the number of training samples. For the* k*NN method, a number of training points equal to the 10% of the volume were not an optimal choice, regardless of the tissue. We experimentally verified that an increase of the number of samples for the small structures leads to a significant improvement. For the caudate, thalamus, pallidum, and putamen, we selected a number of voxels corresponding to roughly the 20% of the volume as optimal parameter. This corresponded to a training size ranging from 500 to 2,000 voxels (corresponding to a volume from 0.5 cc to 2.0 cc at a resolution of 1 × 1 × 1 mm^3^). Moreover, for the* k*NN an unbalanced sample size for the major tissue training has a negative impact on the estimation of some minor structures, since the GM is the tissue with the greater volume and a number of training points proportional to the volume lead to an overestimation of the GM. Experiments revealed that* k*NN works better when GM, WM, and CSF have the balanced number of training samples that was set to 50,000 voxels (corresponding to a volume of 50 cc at a resolution of 1 × 1 × 1 mm^3^).

### 3.2. Segmentation Accuracy

**Figure 3:**
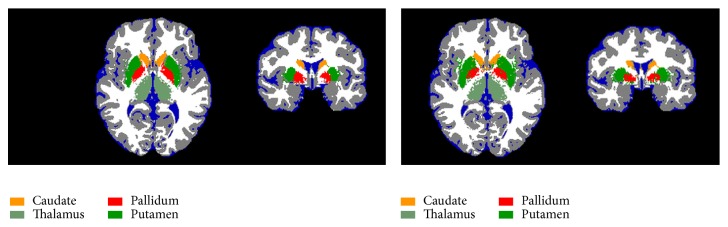
Segmentation results of the automatically trained* k*NN (axial and coronal sections on the left) and PCDA (axial and coronal sections on the right) classifier for one of the studies of the* Image dataset I* (IXI002-Guys-0828).

A visual analysis of the segmentation results for the four subcortical structures was performed for both* k*NN and PCDA methods.  Figure 3 shows the segmented images at the level of basal ganglia for one of the studies of the* Image dataset I*. Classification results are listed in Table 1 which shows the differences in the performance of the two considered methods. For the caudate, thalamus, pallidum, and putamen, the* k*NN exhibits scores from 3 to 5 on the 5-point rating scale with a median value of 4. The PCDA performs worse than* k*NN, with a grade range from 2 to 4 and a median value of 3.

### 3.3. Reproducibility Test

Reproducibility was evaluated by computing the volume differences between scan-rescan imaging sessions. Results are reported in Table 2 for the* k*NN and Table 3 for the PCDA. In the case of the* k*NN algorithm, caudate, thalamus, and putamen were classified with a volume variation less than 5%, for the classifier working with T1 and FLAIR as input, and less than 3.5% when the classifier works with T1, T2, and FLAIR as input. Pallidum shows variability up to 11% in both cases. PCDA exhibits greater instability and less reproducibility in the measurements.

### 3.4. Comparison with an Existing Method

**Figure 4:**
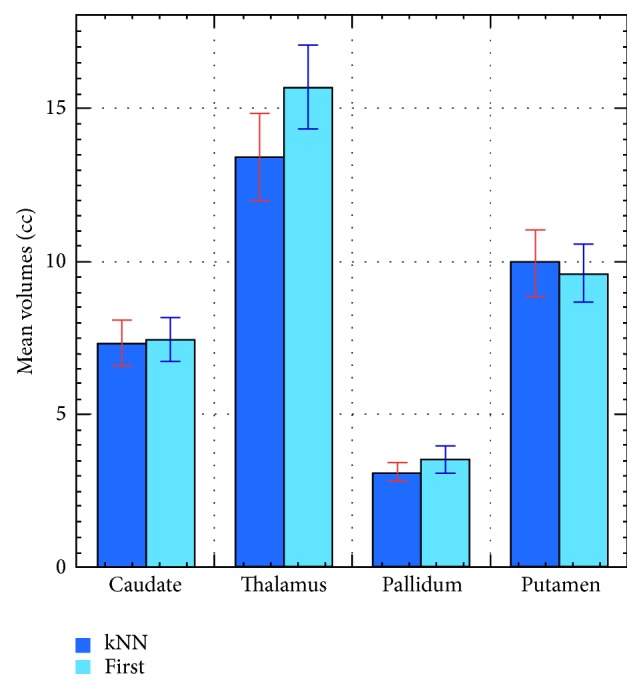
Bar graph showing the mean volume estimates of the automatically trained* k*NN* versus* the FSL-First software for caudate, thalamus, pallidum, and putamen, in the 14 MR studies of the* Image dataset I*. Numerical data are reported in Table 4.

Segmentation results provided by the best performing algorithm (*k*NN) were compared with those obtained with the FSL-First software. Mean volume and the percent of volume overlap have been calculated for caudate, thalamus, pallidum, and putamen, in the 14 MR studies of the* Image dataset I*. Results are summarized in Table 4 and Figure 4. The mean overlap indices were 0.87 for thalamus (range: 0.81–0.90), 0.83 for caudate (range: 0.73–0.88), 0.81 for putamen (range: 0.74–0.86), and 0.76 for pallidum (range: 0.70–0.86).

### 3.5. Execution Times

Both methods considered in this study are computationally efficient. The execution time for the segmentation of a subject is below 8 minutes on a desktop PC with an Intel Core i7 processor, 16 GB RAM, and operating Windows 7.

## 4. Discussion

The objective of this paper is twofold: (1) to study the feasibility of automatically defining a representative training by registering an atlas to the target study and (2) to assess the performance of two atlas-guided trained supervised methods on real MRI data. Training selection is based on thresholded tissue probability atlases coregistered to the subject to define, in intensity and spatial locations, the training dataset of each subcortical structure. The subsequent segmentation by applying supervised methods such as* k*NN and PCDA is in principle feasible for each tissue or structure for which a probability atlas is available. The autotraining is likely applicable to other supervised methods. This is the first study that investigated the ability of automatically trained* k*NN and discriminant analysis methods that combine voxel intensities and spatial coordinates for the classification of subcortical brain structures. A* k*NN classifier trained in a fully automated way using an atlas was initially proposed by Cocosco et al. [9] and Vrooman et al. [11] for the classification of the three major tissues from MR brain images, mainly using intensity information. The combination of intensity and spatial coordinates to be used as features for a* k*NN classifier was instead first proposed by Anbeek et al. [18, 20] and applied to the segmentation of adult and neonatal MR brain images. We focused our attention on the recognition of four subcortical brain structures: caudate, thalamus, pallidum, and putamen. In our approach, the training is not derived on a subset of MR studies with the intent to be performed only once and then used as reference dataset but is always selected on the target study.

A successful supervised segmentation of subcortical structures first requires the definition of a well-founded training of the three main brain tissues in order to derive the corresponding probability density functions; otherwise it will be impossible to differentiate minor structures from underlying tissues. In this study we found that the selection of the training of GM, WM, and CSF on a segmented target study volume achieved with a widely used software (e.g., SPM) allows for obtaining better results than an averaged atlas like ICBM452 coregistered to the subject, providing in addition a superior stability against anatomical variability. Probably, the average and smoothing of the atlas impact the selection of GM, WM, and CSF training samples, leaving the training classes to be nonpure on both spatial location and intensity, thus introducing a bias in the classification process that affects the quality of the segmentation of subcortical structures. For this reason it is important to achieve a reference segmentation of the three main brain tissues in the target study, before starting the subcortical segmentation.

The optimization of the training dataset required an extensive number of tests to select the more appropriate threshold value and the number of samples to be used in the learning phase of the classification process. In particular, for each setting we systematically checked (1) the confusion matrix for the classification of the subvolume formed by the only voxels coincident with the training, (2) the histograms of the intensity features for each tissue, (3) the correspondence between training samples and anatomy (goodness of coregistration), and (4) the final segmented images and the relative volume estimates. Overall, the* k*NN classifier showed a superior stability with respect to small changes in the automatic selection of the training set.

The evaluation of the accuracy of segmentation results in the absence of a reference standard was performed visually by an expert. Results highlighted that caudate, thalamus, pallidum, and putamen received a median rating* good* for the* k*NN, with grades between* fair* and* very good*; for the PCDA the median rating was* fair* with scores ranging from* poor* to* good*.

The reproducibility test, with the limitation to be conducted on a restricted number of studies, showed the best results for the* k*NN method. The tissue with greater variability was the pallidum. The test also highlighted that the* k*NN is able to perform well even with only two contrast types as input, confirming that the essential classification information resides in the T1 and FLAIR channels and consequently that the incorporation of T2 contrast is almost redundant.

The comparison of our results with the well-known and freely available FSL-First software was intended to do a preliminary verification of the agreement of the volume estimates provided by the best performance of the two methods considered in this paper. It should be noted that the approach is different as our segmentation is voxel based while FSL-First defines an enclosed surface shaped to the structure of interest. Table 4 shows that the volumes estimated by the automatically trained* k*NN and the FSL-First software are comparable. Overall, the FSL-First yielded a very good result, with a segmented image less noisy than that provided by* k*NN, although it tends, in some cases, to overestimate the thalamus volume.

The methods considered in this work require multispectral data but not specific acquisition sequences. The experiments conducted on publicly available data acquired in different centers with different scanners and sequences exemplify the generality of the considered approach. The automated training defined by registering an atlas to the target study has been demonstrated to be valid and reliable. This work did not consider the multiatlas approach [25] that hence could be explored as a refinement step in the atlas-guided selection of the training set. The inclusion of additional features for the* k*NN classifier may also be investigated as a possible way to further improve the overall segmentation accuracy.

## 5. Conclusions

Atlas-guided training is a valid and reliable strategy to automatically define a representative training dataset for the segmentation of subcortical structures with supervised methods. Using this training approach, a* k*-nearest neighbor classifier is able to successfully segment caudate, thalamus, pallidum, and putamen from multispectral magnetic resonance brain images without the need of user interaction.

## Figures and Tables

**Algorithm 1:**
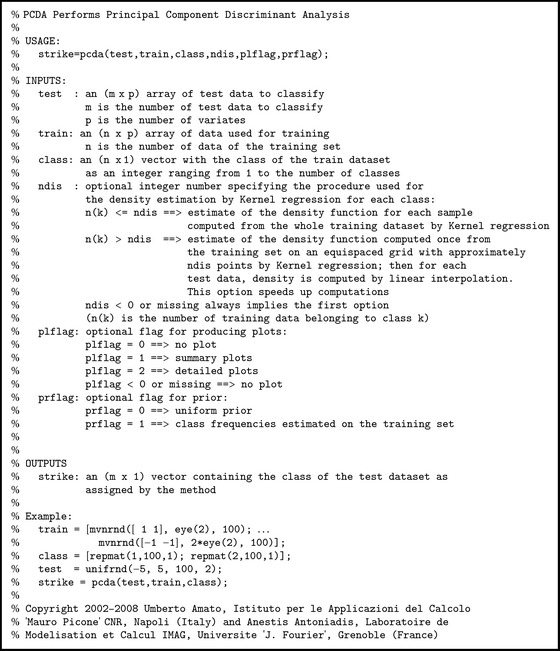


**Table 1:** Evaluation of segmentation algorithms in the 14 MR studies of the *Image dataset I* performed by visual assessment. 1 = very poor, 2 = poor, 3 = fair, 4 = good, 5 = very good; Q1: 1st quartile and Q3: 3rd quartile.

	*k*NN			PCDA		
	Median	Q1	Q3	Median	Q1	Q3
Caudate	4	4.0	4.25	3	2.0	3.0
Thalamus	4	3.0	4.0	3	2.0	3.0
Pallidum	4	3.0	4.0	3	3.0	3.5
Putamen	4	4.0	4.0	3	3.0	3.0

**Table 2:** Results of the reproducibility test for the automatically trained *k*NN algorithm. Volume estimates of the scan and rescan sessions in the case of an input with three and two contrast image types.

		Subject 1			Subject 2			Subject 3		
		Scan (cc)	Rescan (cc)	Δ (%)	Scan (cc)	Rescan (cc)	Δ (%)	Scan (cc)	Rescan (cc)	Δ (%)
*k*NN (T1w, T2w, and FLAIR)	Caudate	8.28	8.18	1.2	6.92	7.02	1.4	7.11	7.08	0.4
	Thalamus	13.58	14.02	3.2	12.87	12.82	0.4	13.98	14.31	2.3
	Pallidum	2.61	2.33	11.3	2.42	2.26	6.8	3.04	2.99	1.7
	Putamen	10.31	10.60	2.8	11.54	11.42	1.0	13.06	12.84	1.7

*k*NN (T1w, FLAIR)	Caudate	9.17	8.92	2.8	7.78	7.97	2.4	8.18	8.17	0.1
	Thalamus	13.49	13.62	1.0	13.04	13.10	0.5	14.42	14.76	2.3
	Pallidum	2.41	2.17	10.5	2.50	2.41	3.7	2.83	2.76	2.5
	Putamen	9.92	10.39	4.6	11.16	11.24	0.7	12.55	12.40	1.2

**Table 3:** Results of the reproducibility test for the automatically trained PCDA algorithm. Volume estimates of the scan and rescan sessions in the case of an input with three and two contrast image types.

		Subject 1			Subject 2			Subject 3		
		Scan (cc)	Rescan (cc)	Δ (%)	Scan (cc)	Rescan (cc)	Δ (%)	Scan (cc)	Rescan (cc)	Δ (%)
PCDA (T1w, T2w, and FLAIR)	Caudate	7.89	7.52	4.8	6.50	6.44	0.9	5.76	7.2	22.2
	Thalamus	13.92	15.63	11.6	12.40	12.38	0.2	12.34	13.75	10.8
	Pallidum	2.17	2.27	4.5	1.74	1.92	9.8	2.47	2.48	0.4
	Putamen	8.41	9.64	13.6	9.89	10.62	7.1	10.79	10.95	1.5

PCDA (T1w, FLAIR)	Caudate	7.19	7.26	1.0	5.57	5.89	5.6	4.52	5.43	18.3
	Thalamus	8.72	10.23	15.9	7.55	8.79	15.2	9.66	11.00	13.0
	Pallidum	1.90	2.12	10.9	2.35	1.92	20.1	2.48	2.44	1.6
	Putamen	6.35	7.06	10.6	7.93	7.87	0.8	8.61	9.05	5.0

**Table 4:** Comparison of subcortical volume estimates provided by *k*NN and FSL-First in terms of mean value and standard deviation for the 14 MR studies of the *Image dataset I*.

	*k*NN		FSL-First	
	Mean (cc)	Std. dev.	Mean (cc)	Std. dev.
Caudate	7.33	0.77	7.45	0.70
Thalamus	13.41	1.43	15.70	1.36
Pallidum	3.12	0.30	3.56	0.45
Putamen	9.96	1.06	9.63	0.93

## Appendices

### A. MR Data Sources

The IXI database (http://biomedic.doc.ic.ac.uk/brain-development/index.php?n=Main.Datasets) is a publicly available collection of nearly 600 MRI scans from normal, healthy subjects.

The fourteen MR studies selected for this work have the following ID: IXI002-Guys-0828, IXI087-Guys-0768, IXI136-HH-1452, IXI143-Guys-0785, IXI176-HH-1604, IXI263-HH-1684, IXI320-Guys-0902, IXI327-HH-1999, IXI351-Guys-0914, IXI499-Guys-1004, IXI562-Guys-1131, IXI567-HH-2536, IXI608-HH-2599, and IXI613-HH-2734.

The Kirby21 database (http://mri.kennedykrieger.org/databases.html) is a publicly available collection of scan-rescan imaging sessions on 21 healthy volunteers with no history of neurological disease. Subjects were imaged twice using the same scanner and acquisition protocol with a complete repositioning of the subject between the first and the second imaging session. The database includes a wide range of MRI sequences as described in [26], from which we selected T1w, T2w, and FLAIR for our segmentation.

The MR studies considered in this work have been downloaded from the NITRC website of Multi-Modal MRI Reproducibility Resource, at http://www.nitrc.org/projects/multimodal.

The six MR studies selected have the following ID: Subject ID 679 (Sessions 03 and 22), Subject ID 913 (Sessions 05 and 31), and Subject ID 492 (Sessions 18 and 38).

### B. Description of the In-House Developed PCDA Function Written in Matlab

See Algorithm 1.
